# Alternative splicing associated with cancer stemness in kidney renal clear cell carcinoma

**DOI:** 10.1186/s12885-021-08470-8

**Published:** 2021-06-15

**Authors:** Lixing Xiao, Guoying Zou, Rui Cheng, Pingping Wang, Kexin Ma, Huimin Cao, Wenyang Zhou, Xiyun Jin, Zhaochun Xu, Yan Huang, Xiaoyu Lin, Huan Nie, Qinghua Jiang

**Affiliations:** 1 Center for Bioinformatics, School of Life Science and Technology, Harbin Institute of Technology, Harbin, 150000 China; 2 Key Laboratory of Biological Big Data (Harbin Institute of Technology), Ministry of Education, Harbin, China

**Keywords:** Alternative splicing, Stemness, Machine learning, KIRC

## Abstract

**Backgroud:**

Cancer stemness is associated with metastases in kidney renal clear cell carcinoma (KIRC) and negatively correlates with immune infiltrates. Recent stemness evaluation methods based on the absolute expression have been proposed to reveal the relationship between stemness and cancer. However, we found that existing methods do not perform well in assessing the stemness of KIRC patients, and they overlooked the impact of alternative splicing. Alternative splicing not only progresses during the differentiation of stem cells, but also changes during the acquisition of the stemness features of cancer stem cells. There is an urgent need for a new method to predict KIRC-specific stemness more accurately, so as to provide help in selecting treatment options.

**Methods:**

The corresponding RNA-Seq data were obtained from the The Cancer Genome Atlas (TCGA) data portal. We also downloaded stem cell RNA sequence data from the Progenitor Cell Biology Consortium (PCBC) Synapse Portal. Independent validation sets with large sample size and common clinic pathological characteristics were obtained from the Gene Expression Omnibus (GEO) database. we constructed a KIRC-specific stemness prediction model using an algorithm called one-class logistic regression based on the expression and alternative splicing data to predict stemness indices of KIRC patients, and the model was externally validated. We identify stemness-associated alternative splicing events (SASEs) by analyzing different alternative splicing event between high- and low- stemness groups. Univariate Cox and multivariable logistic regression analysisw as carried out to detect the prognosis-related SASEs respectively. The area under curve (AUC) of receiver operating characteristic (ROC) was performed to evaluate the predictive values of our model.

**Results:**

Here, we constructed a KIRC-specific stemness prediction model with an AUC of 0.968,and to provide a user-friendly interface of our model for KIRC stemness analysis, we have developed KIRC Stemness Calculator and Visualization (KSCV), hosted on the Shiny server, can most easily be accessed via web browser and the url https://jiang-lab.shinyapps.io/kscv/. When applied to 605 KIRC patients, our stemness indices had a higher correlation with the gender, smoking history and metastasis of the patients than the previous stemness indices, and revealed intratumor heterogeneity at the stemness level. We identified 77 novel SASEs by dividing patients into high- and low- stemness groups with significantly different outcome and they had significant correlations with expression of 17 experimentally validated splicing factors. Both univariate and multivariate survival analysis demonstrated that SASEs closely correlated with the overall survival of patients.

**Conclusions:**

Basing on the stemness indices, we found that not only immune infiltration but also alternative splicing events showed significant different at the stemness level. More importantly, we highlight the critical role of these differential alternative splicing events in poor prognosis, and we believe in the potential for their further translation into targets for immunotherapy.

**Supplementary Information:**

The online version contains supplementary material available at 10.1186/s12885-021-08470-8.

## Backgroud

Renal cell carcinoma is one of the deadliest cancers in the urinary system throughout the world, and its morbidity and mortality are rising rapidly [1]. Kidney Renal Clear Cell Carcinoma (KIRC) is the most common (~ 80%) subtype of renal cancers [2]. About 30% of KIRC patients have metastases at first diagnosis, and 20–40% of patients have recurrence after cancer resection [3]. Although the treatment of KIRC has made progress in the past decade, the mortality rate is still high, especially for patients with advanced/metastatic patients [4]. Metastatic tumor cells spread out from the primary tumor, invade blood vessels, enter the lymphatic and circulatory system [5]. Traditionally, certain pathological stages and grades have been used to predict the prognosis of KIRC patients [6]. However, these methods may be unreliable due to heterogeneity within the patients. Therefore, there is an urgent need for a new method to predict tumor metastasis more accurately, so as to provide help in selecting treatment options.

The most widely used treatment options for tumor cure include surgery, radiation-based surgical knives, chemotherapy, biological treatments, and radiotherapy [7]. Despite the various methods available, a large number of patients continue to relapse after adjuvant therapy, and the survival rate associated with stage IV solid tumors is still very low [8]. Previous studies have shown that this is related to cancer stem cells in cancer tissues. Experimental evidence indicates that a subpopulation of cancer cells, called cancer stem cells, possess “stemness” properties similar to normal stem cells, including self-renewal, differentiation, and proliferative potential [9]. Currently, evidence for the existence of cancer stem cells in a variety of tumors had been growing [10]. Cancer stem cells have the ability of anchorage-independent growth, and they can spread through the blood or lymphatic system to another part of the body, where it grows into a secondary tumor [11]. Cancer stem cells are considered as the source from which tumor cells arise and responsible for metastasis, chemoresistance, and tumor relapse [12]. This hypothesis implies that successful anti-tumor therapy should be based on the elimination or permanent suppression of cancer stem cell. In conclusion, the stemness index used to assess cancer stem cell is an important predictor of cancer metastasis and recurrence time to improve risk assessment and treatment options.

Recently, stemness evaluation methods based on transcription profiles have been proposed to reveal the relationship between stemness and cancer [13]. However, we found that existing tools do not perform well in assessing the stemness of KIRC patients. These predictors are based on the absolute expression value of genes, and do not consider the impact of alternative splicing on cancer. Alternative splicing is an important mechanism in post-transcriptional regulation, and increasing evidences noted that alternative splicing is tightly associated with invasion and metastasis of cancer cells [14]. Alternative splicing is a rich source of tumor-specific neoantigen targets for immunotherapy [15, 16]. It is worth noting that global changes in alternative splicing patterns also occur during the in vitro derivation of embryonic stem cells from the inner cell mass of blastocysts, suggesting that alternative splicing is not only progressing during the differentiation of stem cells, but also during the acquisition of stemness features in cancer stem cells [17].

Here, we constructed a KIRC-specific stemness prediction model using an algorithm called one-class logistic regression based on the expression data and alternative splicing data to predict stemness indices of patients. Based on our stemness indices, we found differences in alternative splicing between high- and low- stemness group in tumors and those stemness-associated splicing events (SASEs) plays a key role in the formation of tumor heterogeneity and poor prognosis which indicates that the stemness indices has potential therapeutic and diagnostic significance and SASEs could serve as biomarkers for KIRC. All of our candidate SASEs may be suitable for further validation and development as therapeutic targets. Our results supported further development of stemness-associated splicing events targeted KIRC-specific therapy strategies, representing an important step forward in therapeutic of KIRC progression.

## Methods

### Data acquisition

The corresponding RNA-Seq data were obtained from the TCGA data portal (https://tcga-data.nci.nih.gov/tcga/). We also downloaded PCBC RNA sequence data from the PCBC Synapse Portal (https://www.synapse.org/pcbc), consisting 16 ESC, 77 iPSC, 66 SC-derived EB, 29 SC-derived MESO, 29 SC-derived ECTO, and 36 SC-derived DE PCBC dataset [13, 18, 19]. Independent clear cell renal cell carcinoma validation sets (GSE73731 [20] and GSE126964 [21]) and stem cell validation set (GSE30652 [22]) with large sample size and common clinic pathological characteristics were obtained from the Gene Expression Omnibus (GEO) database (https://www.ncbi.nlm.nih.gov/geo/).

### Gene expression and alternative splicing differential analysis

RNA-Seq data were analyzed with SpliceSeq software [23] to generate the alternative splicing profiles for each patient as previously described [24–26]. The Percent Spliced In (PSI) value is defined as a percentage of the total (both inclusion and exclusion) normalized read counts for that event. To generate a more reliable set of alternative splicing events, we implemented a series of stringent filters (80% of samples with PSI value, average PSI value ≥0.05). Interactive sets among the seven types of alternative splicing were illustrated by UpSet plot created by UpSetR (version 1.3.3) [27]. To identify KIRC-specific associate alternative splicing events (KASE) in KIRC, the PSI values of alternative splicing events from 39 pairs of KIRC and matched normal tissue were compared. *P*-values were adjusted by Benjamini & Hochberg (BH) correction (|log2FC| ≥ 1, FDR < 0.05, and ΔPSI> 0.1). Differentially expressed genes were identified and visualized by the limma with a threshold of (|logFC| ≥ 1, FDR < 0.05).

### Gene function analysis

Gene Ontology (GO) analyses were conducted for the parent genes of identified KASEs (FDR < 0.05). Function enrichment analysis was performed using the “clusterProfiler” package (version 3.10.1) [28]. Gene set enrichment analysis (GSEA) was performed to verify the differences in biological functions and pathways between tumor and normal tissues identified by clusterProfiler.

### Generation of mRNA stemness indices

To calculate mRNA stemness indices (mRNAsi), we applied OCLR to the pluripotent stem cell samples (which included both ESC and iPSC) to build a predictive model. The score of every stem cell sample was lower than all the non-stem cell samples, yielding an overall AUC of 1.0. We then used the external testing set composed of pluripotent stem cells and somatic cells (229 samples from GSE30652), and the external validation set (66 patients from GSE126964 and 265 patients from GSE73731) for the additional validation of the stemness signature. R packages glmnet and reshape were used as the implementation of this method.

### Identification of stemness-associated splicing events

The degree of stemness for each tumor sample was scored, samples were ranked in ascending order of the mRNAsi. The group of the top 10% samples were similar to stem cells (TSC), and group of the bottom 10% samples were unlike stem cells (USC). To identify stemness-associated alternative splicing events (SASEs) in KIRC, we compared the PSI values between TSC group and USC group. *P*-values were adjusted by Benjamini & Hochberg (BH) correction (|log2FC| ≥ 1, FDR < 0.05, and ΔPSI> 0.2).

### Construction of splicing correlation network

Spearman correlation analysis was performed to explore the association between SASEs and splicing factor expression features. We mapped spliced genes to coding proteins and built the interaction network using Search Tool for Retrieval of Interacting Genes/Proteins (STRING, version 11.0) [29], which was further visualized by Cytoscape (version 3.7) [30].

### Survival analysis

According to the median cutoff of each SASE, KIRC patients were separated into two groups. Univariate Cox regression analysis was performed to calculate 95% confidence interval (95% CI) and hazard ratios (HRs) of SASEs in overall survival. Candidate prognostic SASEs were then subjected to multivariate Cox regression analysis. Kaplan-Meier analysis with log-rank testing was applied to compare survival in different groups. *P*-values were adjusted by Benjamini & Hochberg correction.

### Code availability, statistical analyses and visualization

All statistical analyses were performed in R (version 3.6.3), and *P*-value < 0.05 was considered statistically significant. Student’s t-test and ANOVA test were utilized to compare continuous variables. Survival package for survival analysis. In current work, we employed the CIBERSORT method to evaluate the relative proportions of immune cell profiling [31]. We used ggplot2(3.3.2) and corrplot (0.84) packages for visualization. Spearman’s rank correlation analysis was used for non-normal distribution data. Pearson correlation was used for continuous variables that meet normal distribution.

## Results

### Alternative spliced genes were related to stem cell regulatory pathways in KIRC

Undifferentiated primary tumors are more likely to cause cancer cells to spread to distant organs, leading to disease progression, poor prognosis, and existing therapy resistance [32]. Tathiane M. Malta et al. has provided important information about KIRC stemness [13]. To generate a reliable stemness evaluation index of oncogenic dedifferentiation in KIRC, we ranked the patients in descending order of mRNA stemness indices (mRNAsi) values obtained from Tathiane M. Malta. et.al [13] and stratified patients into high-stemness group (top 10% samples) and low-stemness group (bottom 10% samples). As showed in Fig. S1a, the stemness scores did not match the clinical presentation of patients, as the proportion of high-stemness group in stage II was highest rather than stage IV. KIRC is gender-specific and tends to occur in males [33]. However, the proportions of the high-stemness group and the low-stemness group were almost the same in gender (Fig. S1b). Moreover, smoking was a common carcinogen [34], but contrary to short smoking history, the high-stemness group accounts for a lower proportion of long smoking history (Fig. S1c). These predictors are based on the absolute expression value of genes, and do not consider the impact of alternative splicing on cancer. Accordingly, we found that RNA-expression-based stemness indices do not perform well in assessing the stemness of KIRC patients.

Alterations in alternative splicing also occur during the in vitro derivation of embryonic stem cells from the inner cell mass of blastocysts [17], suggesting that RNA splicing mechanism have been associated with the acquisition of stemness features. As the relationship between spliced genes and stemness in KIRC have not been currently considered, we first decided to a systematic alternative splicing analysis. A total of 605 KIRC patients were identified and the baseline characteristics of these patients are summarized. We preliminarily detected 46,415 alternative splicing events from 10,601 genes. These alternative splicing events were classified into seven splicing modes: alternate acceptor site (AA), alternate donor site (AD), alternate promoter (AP), alternate terminator (AT), exon skipping (ES), mutually exclusive exons (ME) and retained intron (RI), as illustrated in Fig. S2a. Among these splicing modes, ES occurred most frequently (39.0%) and ME were the least (0.51%). The alternative splicing events were screened with a series of filters (80% of samples with PSI value, average PSI value ≥0.05), a total of 34,987 alternative splicing events from 10,205 genes were obtained. After filtering, ES was still the most common mode (41.3%) followed by AP (18.4%) and AT (17.3%) (Fig. S2a). Considering that a single gene may have multiple splicing modes, we created Upset plots to show interactive sets of seven types of alternative splicing events (Fig. S2b). Our results showed a form of genetic regulation of alternative splicing in tumor biology as a single gene coding for different splicing modes may result in dysregulation of multiple proteins. To identify the KIRC-specific alternative splicing events (KASEs), we compared the PSI values between 39 paired tumor and adjacent normal tissues.

**Fig. 1:**
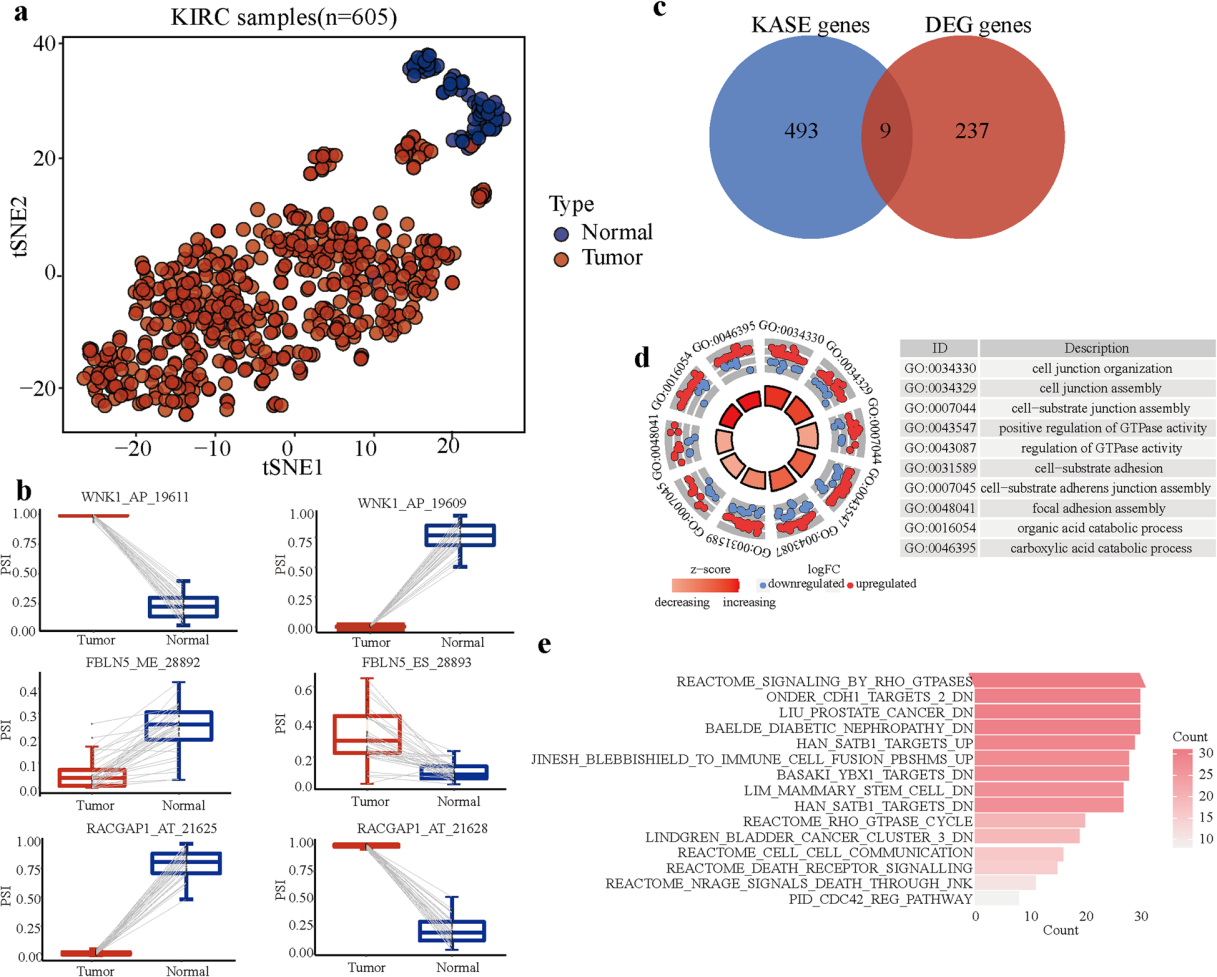
Splicing events in KIRC associated with cancer stemness. **a** tSNE plot of 605 KIRC samples obtained from TCGA and clustered by PSI value of KASE. Cells are colored-coded according to identified tissue types. **b** The PSI value of representative KASEs showing the opposite preference between KIRC and adjacent normal tissues. Student’s t-test was used. *: *P* < 0.05. **c** The intersection of spliced genes and different expression genes. **d** Biological processes analysis of KASEs. The significance was indicated by the adjusted *p*-value (adjusted *p*-value< 0.05) and showed on the height (the curved y-axis) of the red and blue dots indicates. **e** Gene set enrichment analysis of the spliced gene signatures in the Molecular Signatures Database (MSigDB) curated gene sets

A total of 604 KASEs from 502 genes were identified (Supplementary Table 1). Among 78 samples, only two samples were misclassified with an accuracy of 97.4% by hierarchical clustering (Fig. S2c-d). In addition, utilizing t-SNE dimensionality reduction cluster analysis on 605 KIRC samples, KASEs provided the ability to accurately distinguish tumor from normal samples (Fig. 1a). Events related to a single gene, such as WNK1,FBLN5 and RACGAP1, exhibited opposite patterns between tumor and normal samples, indicating that an uneven distribution in the splicing patterns plays different roles in cancer development (Fig. 1b). To further investigate the relationship between alternative splicing dysregulation and gene different expression, we compared the gene expression difference between paired tumor and normal samples (Supplementary Table 2). No more than 2% spliced genes overlapped to that different expression genes which indicated that these dysregulated alternative splicing events are shifts in the balance of alternative splicing, not aberrant splicing which would produce transcripts that are out of frame and undergo NMD (Fig. 1c). Consistent with the effect of alternative splicing independent of gene expression changes in disease progression [35], our results indicated that alternative splicing promoted tumor development independently of gene expression changes in KIRC. As alternative splicing may affect significant domain families in cancers [36], we conducted biological function enrichment analysis of genes related to KASEs.

The results revealed that genes were closely related to the important process regulating the phenotype and function of stem cells, such as cell−substrate adhesion, cell-substrate adherens junction assembly and focal adhesion assembly (Fig. 1d). Pathologically, the loss of cell-cell adhesion molecules in cancer stem cells is thought to contribute to an epithelial to mesenchymal transition and an invasive, migratory phenotype [37, 38]. In addition, we employed the curated gene sets maintained by the Molecular Signatures Database (MSigDB) [39]. Spliced genes have been found in experimentally confirmed gene sets relating to cancer progression and stemness, including the LIM_MAMMARY_STEM_CELL_DN gene set, in which the conserved genes in the mammary stem cell population have been considered as epithelial-mesenchymal transition signature [40], genes and highlights pathways that are likely to govern cell-fate decisions and differentiation (Fig. 1e). Together, spliced genes in KIRC were related to important pathways of regulating stem cells.

### mRNA stemness indices based on spliced genes in KIRC recognized undifferentiated tumors

**Fig. 2:**
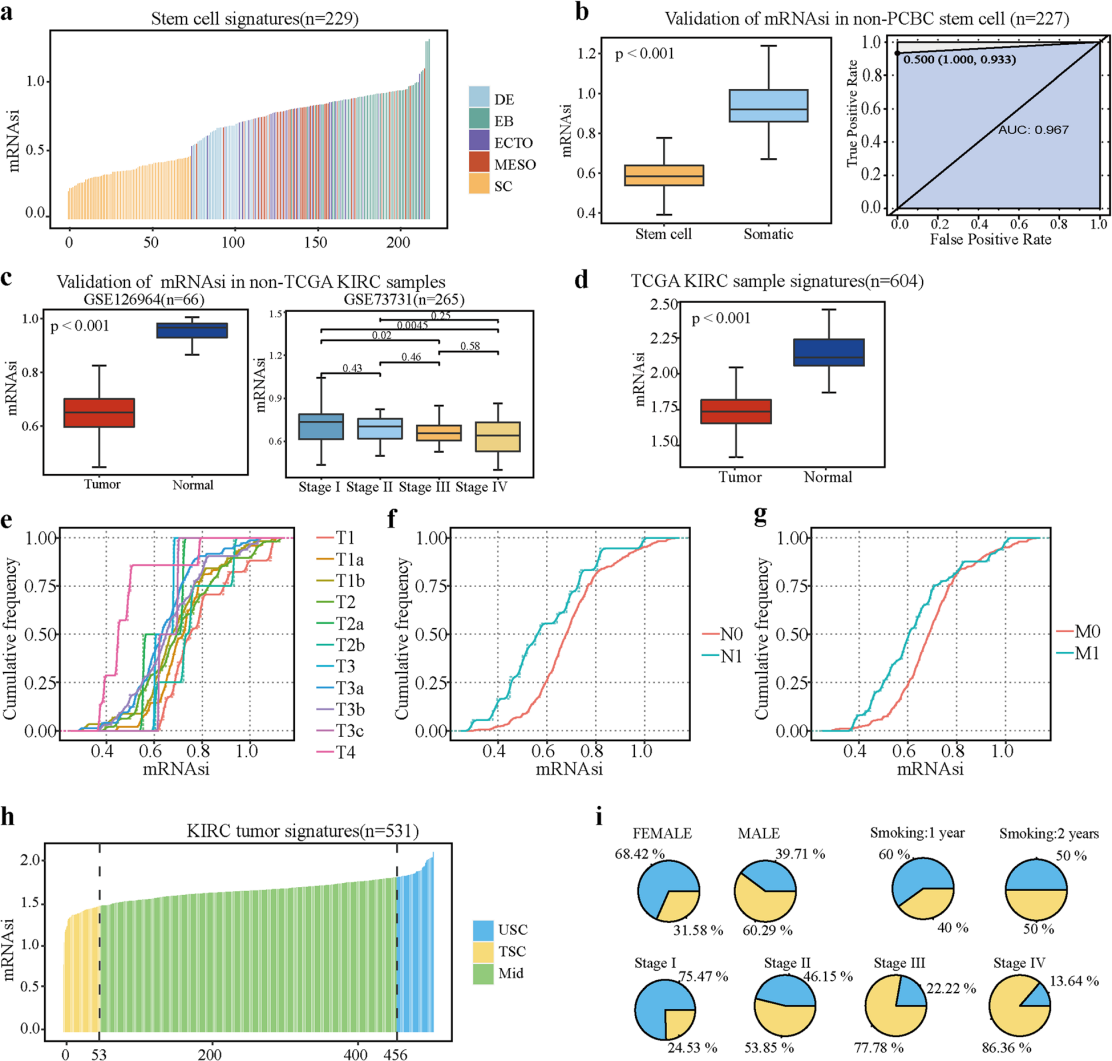
Development and validation of the mRNA stemness indices (mRNAsi). **a** mRNAsi in PCBC stem cell signatures. Stem cell signatures including mRNAsi from endoderm (DE), embryoid bodies (EB), ectoderm (ECTO), mesothelioma (MESO) and stem cell (SC). **b** Stemness indices of the validation set derived using GSE30652 stemness signature. **c** Validation of mRNAsi in non-TCGA kidney cancer (KIRC) samples to define stemness status. Stratification of mRNAsi according to tissue types of GSE126964 samples (left) and different stage of GSE73731 samples (right). **d** Stratification of mRNAsi according to tissue types in TCGA KIRC samples. **e-g** mRNAsi from different tumor progressing (T\N\M stage) of KIRC were compared and showed in ecdf plot. Significance of difference among RCC subtypes were evaluated by Kruskal–Wallis test, *P* value < 0.01. **h** TCGA KIRC tumor types are ranked by mRNAsi; samples are divided into the top 10% samples that are similar to stem cells (TSC), and the middle 10–90% samples (MID), and the bottom 10% that are not similar to stem cells sample (USC). **i** The stemness indices for TSC and USC were correlated with known cancer biology clinical information, such as gender, smoking history and TNM stage

To evaluate the degree of KIRC-specific dedifferentiation considering spliced genes, we constructed a stemness prediction model using the OCLR algorithm trained on stem cell including transformed stem cells and induced pluripotent stem cells categories, and non-stem cells including embryoid bodies, ectoderm, mesothelioma and endoderm (Fig. 2a and Supplementary Table 3). Comparative mRNA stemness indices (mRNAsi) of stem cell and non-stem cell indicated that the undifferentiated samples tended to obtain lower mRNAsi within the model. Additionally, the model was externally validated in 227 stem cell samples (GSE30625) [22] and other two KIRC datasets (66 samples in GSE126964, 265 samples in GSE73731 [20, 21]. Consistent with our previous results that undifferentiated samples scored lower mRNAsi, we distinguished stem cell from somatic cell in a strong capability with an AUC of 0.968 by mRNAsi values (Fig. 2b), and analyses of KIRC samples revealed a tumor samples and advanced clinical stage samples were scored lower mRNAsi (Fig. 2c). We found a negative correlation between tumor progression and stemness indices for the KIRC samples. To provide a user-friendly interface of our model for KIRC stemness analysis, we have developed KIRC Stemness Calculator and Visualization (KSCV), hosted on the Shiny server, can most easily be accessed via web browser and the url https://jiang-lab.shinyapps.io/kscv/.

Our model was then applied to the entire TCGA KIRC dataset and the tumor samples were showed in relatively lower mRNAsi scores, indicating that undifferentiated KIRC tumors were inclined to be in a similar mRNAsi trend to that of stem cells (Fig. 2d and Supplementary Table 4). To clarify the relationship between mRNAsi and tumor metastasis, mRNAsi from different tumor progressing (T\N\M stage) of KIRC were compared and showed in ecdf plot. Compared with early neoplasm, such as T4/N0/M0 stage, samples in metastatic tumors had relatively lower mRNAsi, which consistent with our previous result that lower mRNAsi correlated with higher stemness (Fig. 2e-g).

To include direct comparisons of model from Malta et al.(model1) and our model (model2) for discriminating between KIRC versus healthy, we analyzed the distribution of mRNAsi from the two models between normal and tumor tissues. We found that model2 separated normal and tumor tissues better, while the mRNAsi of model1 showed a large degree of overlap between them. In addition, the mRNAsi of tumor group in model 1 was lower than that of the normal group, which was inconsistent with the higher correlation of mRNAsi with malignant cells in previous study [13]. In model2, stem cell samples attained lower si values than samples from differentiated cells, which indicates that model2 correctly identified the stem cell characteristics of the tumor samples with lower mRNAsi (Fig. S3a). Comparing with the mRNAsi of model 1 which has no significant trend, the mRNAsi of model2 gradually decreased with the progress of cancer, and showed better consistency in evaluating the health of KIRC (Fig. S3b). We performed correlation analysis on mRNAsi, stage and overall survival (OS) from model1 and model2. We found that there was no significant correlation between mRNAsi from model1 and model2, but mRNAsi from model2 was related to the stage and OS of KIRC (Fig. S3c). According to the median value of mRNAsi, we divided the samples into high and low groups. We found that mRNAsi from model1 was not associated with prognosis and model2 was more capable of predicting prognosis than model1(Fig. S3d).

Basing on the ascending sorting of mRNAsi score, the KIRC tumor samples were subtyped into two groups: group of the top 10% samples in were similar to stem cells (TSC), and group of the bottom 10% samples were unlike stem cells (USC) (Fig. 2h). Compared with the USC group, the proportion of TSC in male is higher (Fig. 2i). It is well know that smoking is positively associated with cancer development [34]. Therefore, we analysis the proportion of TSC and USC in different smoking history. We found that the proportion of TSC increased with smoking history (Fig. 2i). In addition, the proportion of TSC from advanced cancer were growing as TSC accounted for 86.36% in stage IV, while contrasted with that was less than 25% in stage I (Fig. 2i). Our results suggest that splicing gene based mRNAsi has reliable clinical utility in predicting malignant KIRC progression.

### Stemness-associated alternative splicing events contributed to tumor heterogeneity

**Fig. 3:**
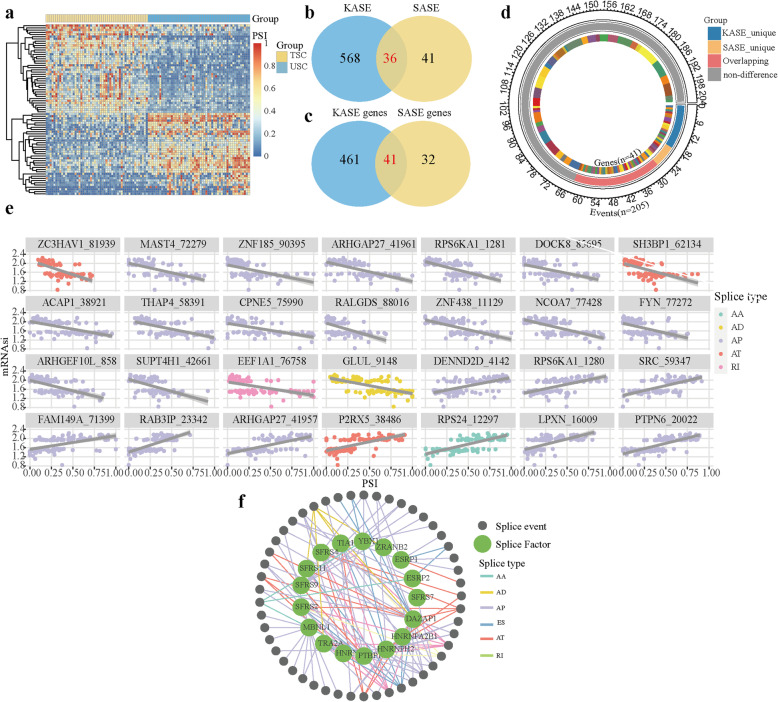
Identification of SASE in KIRC. **a** Heatmap of the SASEs between TSC group and USC group (|log2FC| ≥ 1, adjusted *P* < 0.05 and ΔPSI> 0.1). **b-c** Intersection of events and genes between KASEs and SASEs. **d** Circos plot of the annotation of intersected genes in (**c**) and their related splicing events. The outer circle is composed of the polyline that represents the number of splicing events generated by the intersected genes. The intermediate circle represents the group of splicing events, including events unique to KASEs, events unique to SASEs, events overlapped in KASEs and SASEs, and events that were not a significant different event. The inner circle represents the intersected genes in (**c**). The ribbons represent the 41 genes. **e** Correlation between mRNAsi and PSI values of SASE. **f** Correlation network of splicing factors and SASEs in KIRC. The splicing correlation network was built based on significant correlations between the PSI values of SASEs and the expression of splicing factors. A node represents an SASEs or a splicing factor, which is distinguished by color of the node. The color of lines represents splicing types

A growing body of evidence demonstrates that dysregulation of alternative splicing events can function as biomarkers and therapeutic targets for diverse types of cancers [41]. The specificity or severity of cancer-associated splicing events was demonstrated to facilitate sensitivity to spliceosome-targeting therapy [42]. To unravel the intricate relationship between alternative splicing and tumor heterogeneity in KIRC, we identified the stemness-related alternative splicing event (SASEs) between TSC group and USC group, and 77 events were found in significant difference in alternative splicing (Fig. 3a and Supplementary Table 5). We found far fewer SASEs than in our previous differential splicing analysis about KASEs. To determine the splicing events that only differ between tumors, we compared events and genes related to KASE and SASE respectively. In Venn diagram, there were 36 events intersected in KASEs and SASEs, and more than a half of events were unique in SASE (Fig. 3b). Since these events in SASE were only different in TSC and USC, our results indicated that SASEs contributed to tumor heterogeneity and as the stemness increased, the number of abnormal alternative splicing events in tumors decreased which may result in a reduction in the number of antigens produced. Interestingly, there were 41 intersecting genes related to KASEs and SASEs, which was more than that 36 intersected events (Fig. 3c). This was contrary to the prior knowledge that gene generated multiple events. Exploring the composition of the intersecting genes, we observed that 206 events were generated by these genes in KIRC. In addition to 36 overlapping events in KASEs and SASEs, there were events unique in KASE and SASE separately, and part of events were not significant different in alternative splicing (Fig. 3d). We found that SASEs showed tumor heterogeneity and revealed the dynamic changes of alternative splicing in the tumor development in KIRC.

To extend our findings to the potential mechanisms of SASEs, we performed correlation analysis between PSI values and mRNAsi scores (FDR < 0.05, |R| > 0.6). As shown in Fig. 3e, there were 28 events highly related to stemness, and among them 18 events have negatively related to mRNAsi which indicated that inclusion of pre-mRNA was inclined to occur in undifferentiated tumors. In keeping with previous research showing splicing factors regulated pre-mRNA splicing [41], we found that expression of 17 experimentally validated splicing factors had significant correlations to the PSI values of SASEs (FDR < 0.05, |R| > 0.6), and a splicing regulatory network was constructed (Fig. 3f). Most splicing factors were significantly related to more than one SASE. In addition, one SASE was regulated by multiple different splicing factors, which reflected the complex cooperation and competition between splicing factors. To further verify the regulation of splicing factors on SASEs, we investigated the protein-protein interaction network of splicing factors to provide the interactions in the normal state at the protein level (Fig. S4a). Notably, the expression of splicing factor ESRP1, regulating alternative splicing in epithelial cells [43], was also correlated with DNA methylation levels (Fig. S4b). Correlation analysis revealed that higher expression of SASEs was associated with CD8 T cell infiltration (Fig. S4c). Together, there was intratumoral heterogeneity in stemness-related alternative splicing, suggesting splicing factors further regulate abnormal alternative splicing events.

### Clinical relevance of stemness-associated splicing events

**Fig. 4:**
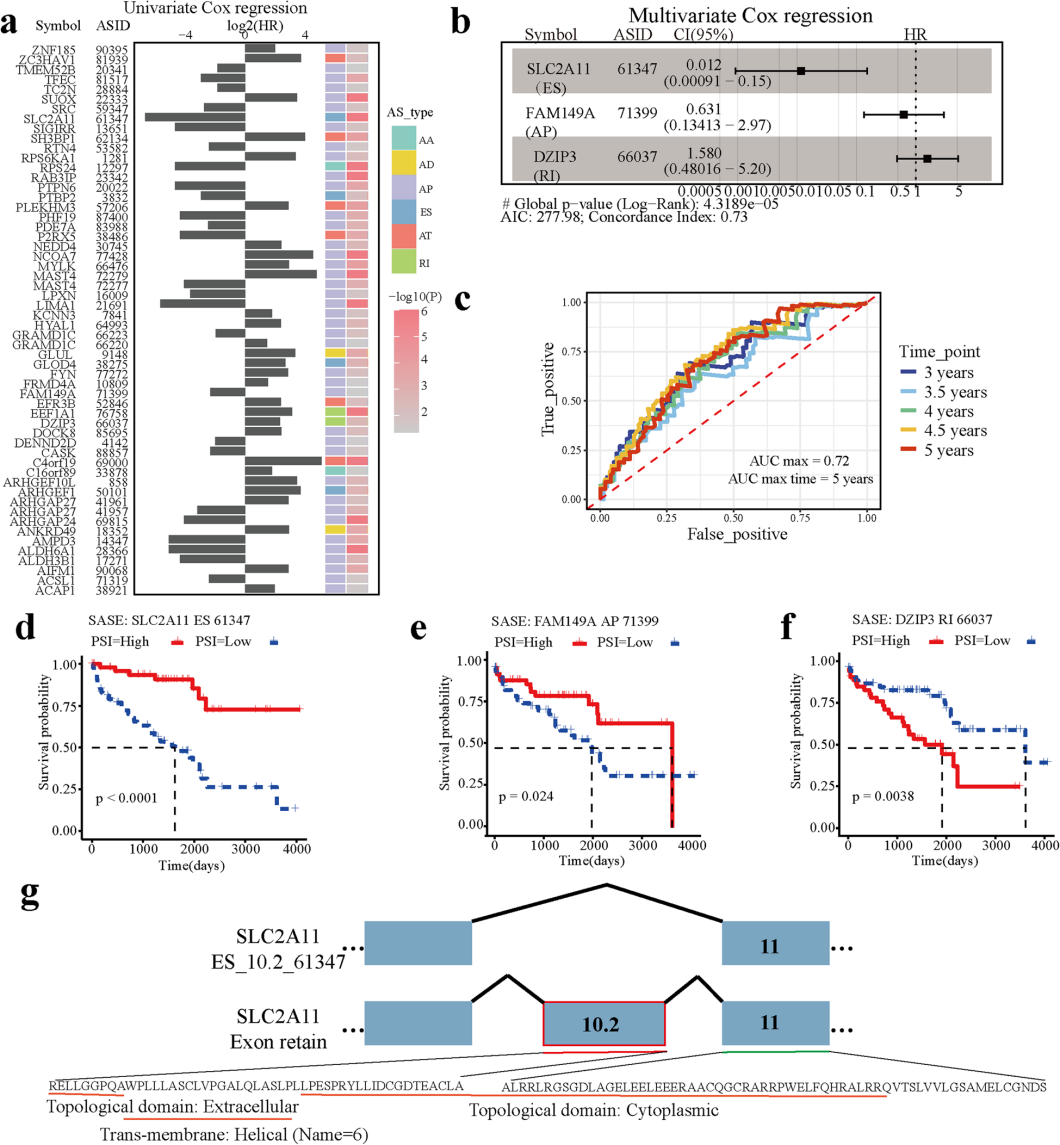
Potential transcription factors essential for KIRC cancer progression. **a** Hazard ratios and 95% confidence intervals of hazard ratios of transcription factors in relation to overall survival. **b** LASSO Cox regression coefficients of transcription factors in relation to overall survival. **c** ROC (receiver operating characteristic) curve analysis. Comparison of ROC curves between different time. **d-f** Overall survival associated SASEs (*p*-value < 0.05). Samples in (**d**) *SLC2A11*_ES_61347 set, (**e**) *FAM149A*_AP_71399 set, (**f**) *DZIP3*_RI_66037 were dichotomized by PSI into a high group and a low group. The two groups were compared by Kaplan-Meier curves, and the *P*-value was calculated by log-rank test. **g** Isoforms and proteins generated by *SLC2A11* affected the functional domains

Targeted therapy is a cancer treatment that uses drugs to target specific genes and proteins related to the growth and survival of cancer cells. However, drugs that are different from and not on its target biological target may cause off-target activity, which is the most common contributes to side effects [44]. Therefore, further studies are needed to ensure a full understanding of their mechanisms of action. Alternative splicing widely occurs in tumor samples and it has been proven to contribute to the generation of candidate neoantigens [15]. Here, we found that KIRC has shown a variety of specificities in alternative splicing. To determine the relationship between SASEs and prognosis, we ran univariate Cox regression and multivariable logistic regression. Using univariate Cox proportional hazards regression analysis (Hazard Ratio (HR) ≠ 1, *p* < 0.05), we observed that more than 70% of SASEs related to prognosis (Fig. 4a). Then a multivariate Cox model was performed to describe the risk factors associated with 3- to 5-year survival (Global *p*-value< 0.05; Fig. 4b). Our result showed that the lower PSI value of SLC2A11_ES_61347 and FAM149A_AP_71399 (HR < 1) indicated poor prognosis of patients, and the higher PSI value of DAIP3_RI_66037 related to poor prognosis. The areas under the ROC curve were 0.72 (4.5-year ROC) and 0.702 (5-year ROC), and the C-index was 0.73 (Fig. 4c). Both univariate and multivariate survival analysis demonstrated that SASEs closely correlated with the overall survival of patients (Fig. 4d-f). Moreover, RNA-seq data from SLC2A11, a novel sugar transporter [45], showed that the tendency of TSC to skip 10.2 exon resulted in the complete domain loss of extracellular and helix (name = 6), and the partial domain deletion of cytoplasmic (Fig. 4g). Cumulatively, these results suggested that splicing events related to stemness may serve as a new prognostic marker for KIRC. All of our candidate SASEs would be suitable for further validation and development as therapeutic targets.

## Discussion

In this study, we revealed previous stemness indices based on the absolute expression value of genes do not perform well in assessing the stemness of KIRC patients. Here, a KIRC-specific stemness prediction model based on the expression and alternative splicing data with good prediction accuracy was constructed to predict stemness indices of patients. Basing on the stemness indices, we found differential alternative splicing events in tumors at stem level. Moreover, these splicing events are involved in poor prognosis and may become potential immunotherapy targets in tumors. This study suggests the importance of alternative splicing in cancer stemness.

The abnormal regulation of alternative splicing is usually accompanied by the occurrence and development of tumors, which would produce multiple different isoforms and diversify protein expression [46, 47]. This may impact the process of feature selection. Comparing with previous stemness indices, this is the first study to develop KIRC-specific stemness indices based on alternative splicing and expression data. When applied to 605 KIRC patients, our stemness indices had a higher correlation with the risk factors of the patients than the previous stemness indices and revealed intratumor heterogeneity at the stemness level. Previous studies have shown that tumor metastasis is related to cancer stemness [48]. Especially, our stemness indices were highly correlated with the possibility of patient metastasis, so we can determine the tumor grade and provide help in choosing treatment options.

Many studies show have shown that there are alternative splicing differences between tumor and normal tissues [42]. However, studies on alternative splicing between different stemness tumor tissues are still lacking. Here, we identified 77 significant different splicing events between high- and low- stemness groups in tumor samples, and more than 70% of them related to prognosis and were intricately regulated by splicing factors network. Recent study described that alternative splicing contributed to the generation of candidate neoantigens [49], and a negative association between cancer stemness and immune infiltrates has also been proved [50]. Our results provide new insights into immune infiltrates at stemness level. We found that as the stemness increased, the number of abnormal alternative splicing events in tumor decreases, resulting in a decrease in the number of antigens produced, which was related to the change of immune infiltration during cancer progression. Changes in the cancer stemness lead to differences in alternative splicing, among which the expression of splicing factor serve as important influence [51, 52]. Splicing factor expression in tumor greatly affected the alternative splicing, leading to the decrease of neoantigens and thus the decrease of immune infiltration [53]. Therefore, further studies on the stemness-associated splicing events are needed to ensure a full understanding of their mechanisms of action.

Alternative splicing events serve as an important biomarker in cancers [54], and we found *SLC2A11*_ES_61347, *FAM149A*_AP_71399 and *DZIP3*_RI_66037 provided effective and stable prognosis-targeted marker. Notably, kidney has a key role in maintaining glucose homeostasis, and SLC2A11 as a novel, muscle-specific transport facilitator is a member of the extended GLUT family of sugar/polyol-transport facilitators, and this may be an additional source of energy for cancer [55, 56]. GLUT can be also a target-specific therapy as an anticancer therapy [57]. Based on the obtained results, we postulate that *SLC2A11*_ES_61347 may be correlated with tumor differentiation and may play a role in KIRC development.

In summary, our KIRC-specific stemness prediction model performs well in predicting the cancer stemness, and is reliable to predict the metastasis of cancer which guides therapeutic targeting of the cancer stemness. In particular, we found stemness-associated splicing events play a causative role in the formation of tumor heterogeneity, it may be beneficial to target specific molecules or pathways for cancer neoantigens or immunotherapy.

## Conclusions

Basing on the stemness indices, we found that not only immune infiltration but also alternative splicing events showed significant different at the stemness level. More importantly, we highlight the critical role of these differential alternative splicing events in poor prognosis, and we believe in the potential for their further translation into targets for immunotherapy.

## Supplementary Information

**Additional file 1.**

**Additional file 2.**

## Data Availability

All the data applied in the present study were obtained from the publicly available databases: TCGA data portal (https://tcga-data.nci.nih.gov/tcga/). PCBC Synapse Portal (https://www.synapse.org/pcbc). Independent validation sets (GSE73731, GSE126964 and GSE30652) were obtained from the Gene Expression Omnibus (GEO) database (https://www.ncbi.nlm.nih.gov/geo/).

## Abbreviations

TCGA: The Cancer Genome Atlas (TCGA) data portal.
PCBC: The Progenitor Cell Biology Consortium
GEO: The Gene Expression Omnibus
KIRC: Kidney renal clear cell carcinoma
SASE: Stemness-associated alternative splicing events
PSI: Percent Spliced In
TSC: Samples were similar to stem cells (TSC)
USC: Samples were unlike stem cells (USC)
mRNAsi: mRNA stemness indices
AUC: The area under curve (AUC)
ROC: Receiver operating characteristic
