# Supplementary material for: Alternative splicing associated with cancer stemness in kidney renal clear cell carcinoma
Source: BMC Cancer. 2021 Jun 15;21:703. doi: 10.1186/s12885-021-08470-8 (PMC8204412; doi:10.1186/s12885-021-08470-8)
Supplement: Supplementary file 1 — Additional file 1. [file 12885_2021_8470_MOESM1_ESM.pdf]

Alternative splicing associated with cancer stemness in kidney renal clear cell carcinoma

Lixing Xiao[1#], Guoying Zou[1#], Rui Cheng[1#], Pingping Wang[1], Kexin Ma[1], Huimin Cao[1], Wenyang Zhou[1], Xiyun Jin[1], Zhaochun Xu[1], Yan Huang[1], Xiaoyu Lin[1], Huan Nie[1*], Qinghua Jiang[1,2*]

[1]Center for Bioinformatics, School of Life Science and Technology, Harbin Institute of Technology, Harbin 150000, China

[2]Key Laboratory of Biological Big Data (Harbin Institute of Technology), Ministry of Education, China

# These authors contributed equally to this work.

*To whom correspondence should be addressed: Qinghua Jiang (Email: qhjiang@hit.edu.cn), Huan Nie (Email: nh1212@hit.edu.cn)

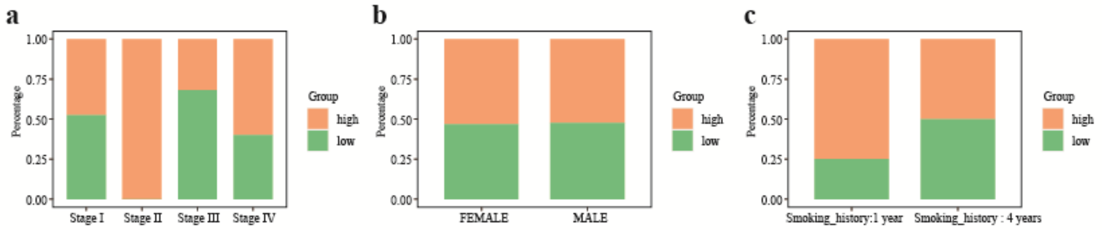

Supplementary Figure 1. Validation mRNAsi from previous study in clinical features.

mRNAsi from previous study was validated in clinical features, including TNM staging (a), gender (b), and smoking history (c).

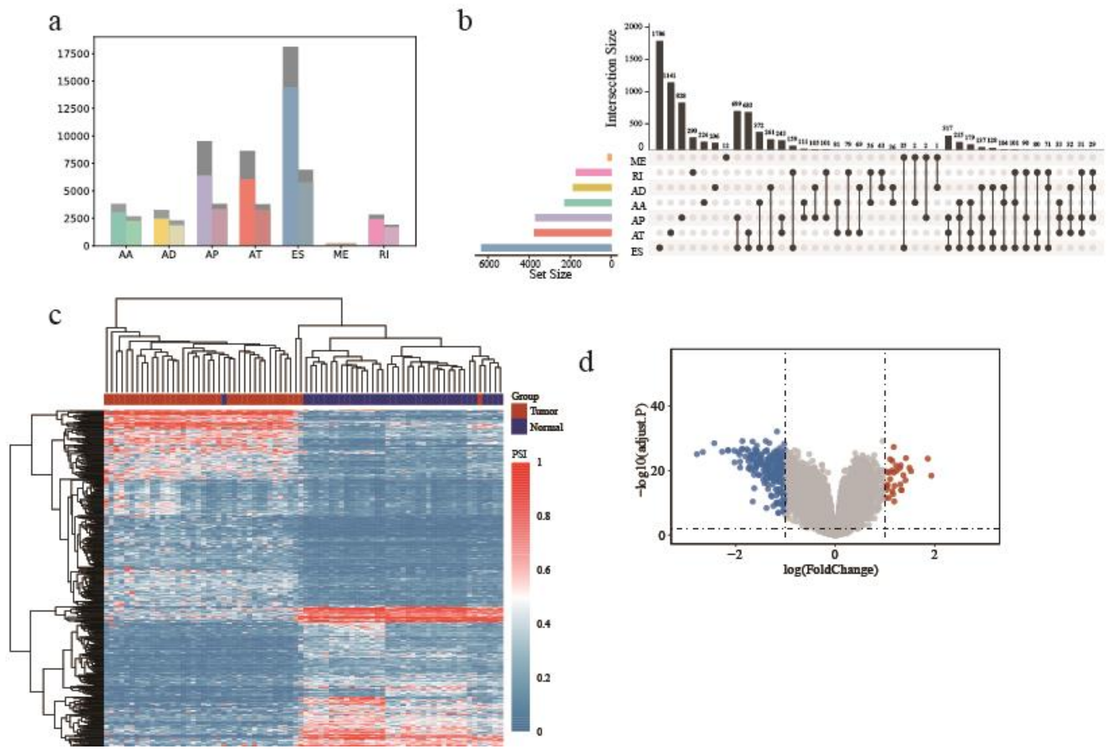

Supplementary Figure 2. Profiling of integrated splicing events detected in KIRC.

(a)Number of splicing events and their related genes in the KIRC patients. the splicing events and their related genes using stringent filters were colored. The grey bars represent the splicing events and their related genes before data progressing. (b) Splicing modes of filtered splicing profile shown in an UpSet plot. (c) Heatmap of the KASEs between 39 paired tumor and normal tissues ($|log2FC| \geq 1$, adjusted $P < 0.05$). (d) Volcano plot of KASEs identified in KIRC. The red and blue points in the plot represent up regulated and down regulated KASEs, respectively.

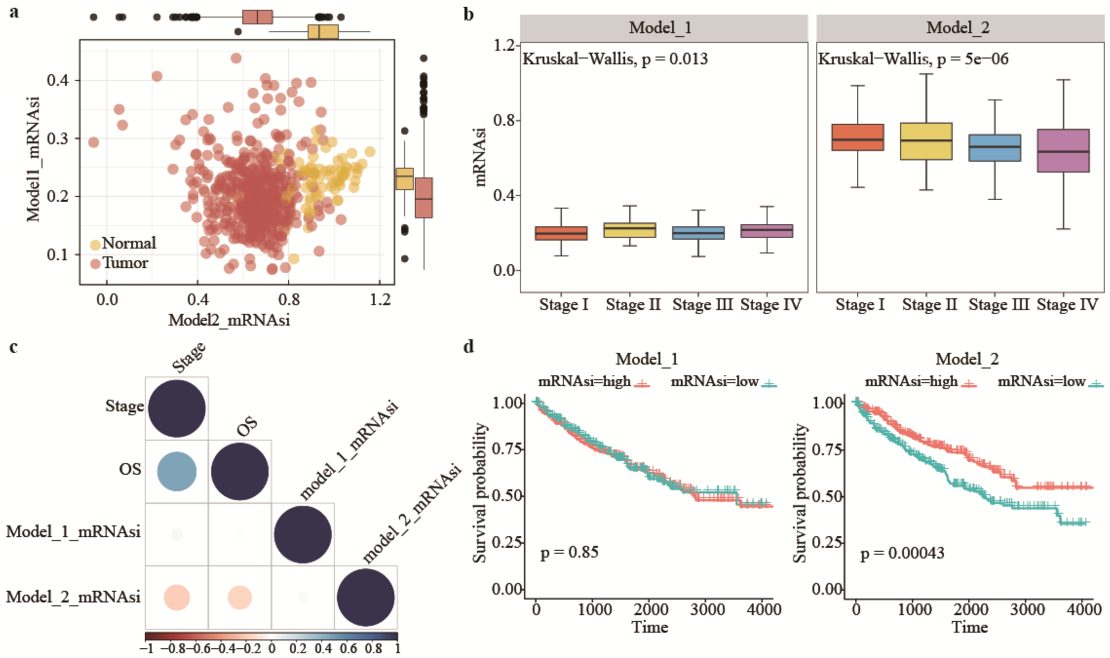

Supplementary Figure 3. Comparisons of the gene-expression model (model1) and the genes and alternative splicing events model (model2). (a) Scatter plots and box plots show detailed information about the distribution of mRNAsi (x-axis: model1; y-axis: model2) in normal and tumor samples. (b) The mRNAsi from model1 (left) did not show a clear trend, while the mRNAsi from model2 (right) was gradually decreasing in the malignant progression of KIRC. (c) Correlation of mRNAsi from model1 and model2, stage and overall survival (OS). Positive correlations are shown in blue, and negative correlations are shown in red. The color intensity and the size of the circle are proportional to the correlation coefficient. (d) The samples were divided into high and low groups by median mRNAsi from model 1 (left) and model 2 (right) separately. The two groups were compared by Kaplan-Meier curves, and the P-value was calculated by log-rank test. Survival analysis showed that model2 has a better ability to predict prognosis than model1.

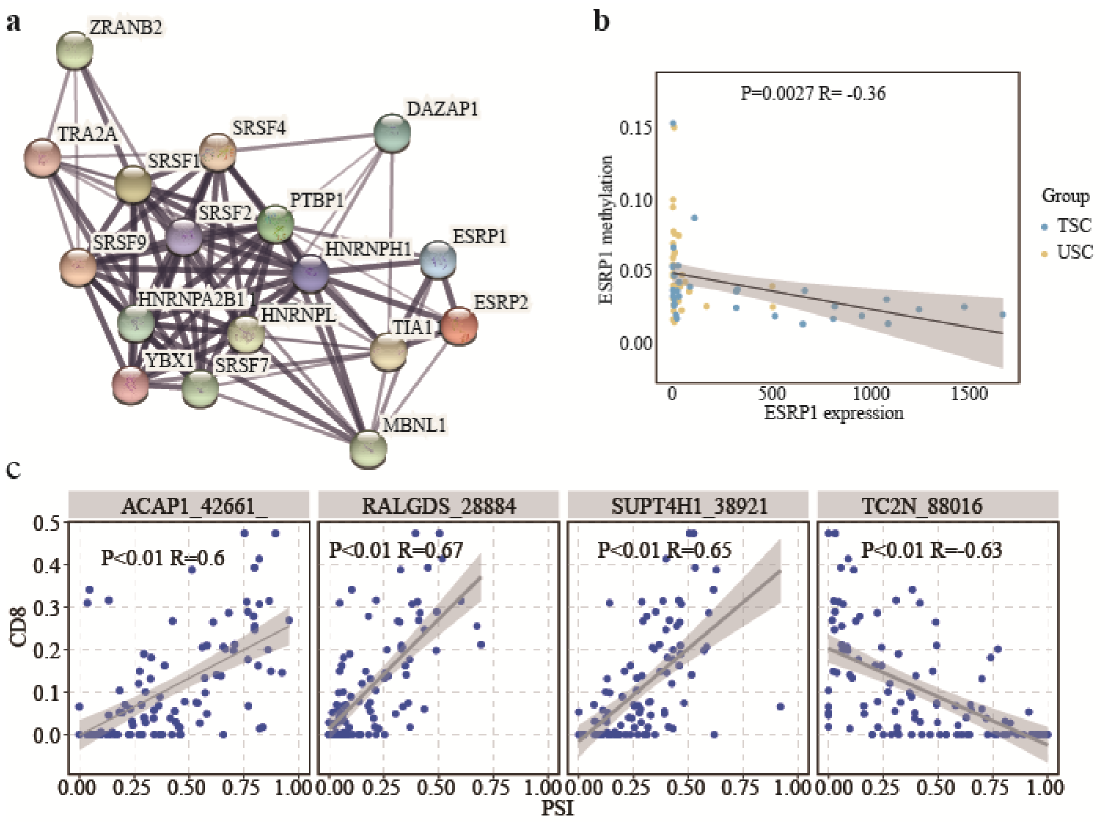

Supplementary Figure 4. Potential biological function of SASEs.

(a) PPI network of splicing factors related to SASEs was generated by Cytoscape. Nodes represent splicing factors. (b) Correlation between expression and methylation values of ESRP1. (c) Correlation analysis of specific SASEs and CD8+ T-cell infiltration. Adjusted $P < 0.05$.

Supplementary Table 1 | KIRC-associated alternative splicing events

Supplementary Table 2 | Different expression genes in KIRC

Supplementary Table 3 | mRNAsi of stem cell signatures from PCBC

Supplementary Table 4 | mRNAsi of TCGA KIRC samples

Supplementary Table 5 | Stemness-associated splicing event in KIRC
